# Club Drugs and Psychiatric Outcomes: A Descriptive Case Series from Spain

**DOI:** 10.3390/ph17101387

**Published:** 2024-10-17

**Authors:** Chiara Montemitro, Alessio Mosca, Stefania Chiappini, Andrea Miuli, Fabrizio Schifano, Maria Josè Gordillo Montano, Cristina Merino del Villar, Rita Allegretti, Carlotta Marrangone, Gilberto Di Petta, Domenico De Berardis, Mauro Pettorruso, Giovanni Martinotti

**Affiliations:** 1 Department of Neurosciences, Imaging and Clinical Sciences, G. D’Annunzio University of Chieti, 66100 Chieti, Italy; chiara.montemitro@gmail.com (C.M.); stefaniachiappini9@gmail.com (S.C.); carlotta.marrangone@gmail.com (C.M.);; 2 School of Medicine, UniCamillus International Medical School University, Via di S. Alessandro 8, 00131 Rome, Italy; 3 Psychopharmacology, Drug Misuse and Novel Psychoactive Substances Research Unit, School of Life and Medical Sciences, University of Hertfordshire, Hatfield AL10 9AB, UK; 4 Hospital Can Misses Hospital, 07800 Eivissa, Spain; 5 Mental Health Department, Santa Maria delle Grazie Hospital, ASL 2, 80078 Naples, Italy; 6 Department of Mental Health, ASL, 64100 Teramo, Italy; domenico.deberardis@aslteramo.it

**Keywords:** NPS, new psychoactive substances, club drugs, drug abuse, drug misuse, substance-induced psychosis

## Abstract

Background: illegal drugs significantly contribute to global health issues, with health complications often occurring not only in regular users with Substance Use Disorders (SUDs) but also in first-time and occasional users. Methods: this study examines five clinical cases from a public hospital in Ibiza, Spain, where patients presented with acute psychiatric symptoms due to recreational drug use. Results: Contrary to previous studies on SUDs, our patients typically had higher education levels and stable employment. Most of them used multiple substances, with cannabis, cocaine, and alcohol being the most frequently used. There was also a common occurrence of consuming drugs with uncertain contents. Upon admission, typical symptoms included aggression, hallucinations, mood swings, and disorientation in time and space. Conclusions: Our findings underscore the significant mental health risks posed by illicit drugs, even for individuals with no prior psychiatric history. Factors like the drug’s potency, frequency and amount of use, past mental health issues, personality traits, and previous traumatic experiences might influence the onset of these symptoms.

## 1. Introduction

Illicit drug use significantly contributes to the global disease burden. Notably, access to psychiatric or emergency departments is not limited to patients diagnosed with Substance Use Disorders (SUDs) [1]. Occasional and recreational drug users may experience complications similar to the ones reported in regular users and need medical attention. The physical and psychological risks associated with occasional drug use are often underestimated. Although psychiatric disorders and SUDs may often coexist, leading to a group of conditions known as ‘dual diagnoses’, the underlying causes of these extremely high comorbidity rates remain mostly unidentified despite various theories [2,3].

Recreational drugs, which can be legal, controlled, or illegal, are substances taken for personal enjoyment rather than medical needs. Alongside traditional drugs, new psychoactive substances (NPSs) continuously enter the drug market, increasing health concerns [4,5,6,7,8,9,10,11,12,13,14,15]. NPSs, as defined by the European Union, refer to those drugs that might not be regulated under existing conventions but still pose significant health risks [16]. These include substances like synthetic cannabinoids (e.g., Spice), cathinones, phenethylamines, synthetic opioids, designer benzodiazepines, etc. The health risks tied to NPS use, ranging from aggressive behaviors to psychotic symptoms, are often underestimated by both consumers and medical professionals [16,17,18,19]. Notably, NPS-induced fatalities, primarily studied in the United Kingdom (UK) [20,21,22,23], seem prevalent in areas with active nightlife, like the Balearic Islands.

In this context, emergency and psychiatric care departments frequently attend to individuals who do not necessarily have a diagnosis of a SUD, but they exhibit sporadic recreational drug use [24] or experimented drugs for the first time [25]. Tourist hotspots—like Ibiza—with their vibrant nightlife, become a crucial focus for addressing the risks associated with drugs and especially NPSs. Some studies suggest tourists in Ibiza engage in highly risky behaviors more than matched controls in the UK, with young tourists and foreign workers being the mostly engaged in excessive alcohol consumption, substance abuse, and risky sexual activities [26]. There are also tales of drug traffickers exploiting tourists by offering potent, dangerous NPS variants for trial [27,28,29]. This is not surprising, considering the extensive literature showing higher risks for drug use in young club-goers compared to their non-clubbing counterparts [30,31,32]. The surge in international nightlife tourism, combined with the challenges of navigating unfamiliar drug terrains in foreign lands, demands internationally coordinated health and safety interventions [33].

In light of the aforementioned context, we aim to present findings from a series of cases collected in Ibiza during the summer clubbing season. Our aim was to explore possible relationships between club drugs exposure and the onset of severe psychiatric symptoms that require medical attention and hospitalization.

## 2. Results

Here, we present the clinical history of five patients admitted to the Psychiatry Department of the Can Misses Hospital after clubbing on the island. Our findings have been summarized in Table 1.

### 2.1. Case 1

A 27-year-old Norwegian woman was admitted after a psychotic episode with paranoid features. She was spending the summer holidays in Ibiza with some friends, when she started feeling threatened and scared. She was convinced that someone (probably her ex-boyfriend) had stolen her papers and identity card, preventing her from returning to Norway.

On the day of the admission, she presented with psychomotor agitation, verbal and outwardly directed aggression, emotional lability, and conceptual disorganization. Physical restraint was not necessary; olanzapine, haloperidol, and lorazepam were administered. Any previous access to psychiatric services was denied, as well as any psychiatric history. The patient also denied any substance use. She claimed to be a student, occasionally hired for temporary jobs. However, because of the high levels of hostility and paranoia, the patient was initially not accessible for a full clinical interview. She refused to answer any questions, appeared uncomfortable with the medical staff whom she evidently did not trust; she was concerned about drug therapy and felt that she was being used as a guinea pig by the medical staff, experimenting new substances. She appeared extremely distressed by the situation. On the day of hospital admission, speech was spontaneous, but disorganized; there was evidence of emotional lability and inconsistency between facial expression and speech content, psychomotor arousal, irritability, and a tendency towards verbal aggression. Also, she was seemed disoriented in both space and time, with conceptual disorganization and persecutory delusional ideation towards the medical staff, and she often asked the interviewer to return her documents. No insight nor perception of illness was detected. The next day, a lowered mood and the effects of delayed insomnia, a lack of energy and spatio-temporal disorientation, confusion, and mannerisms were observed. She spoke of a boyfriend, currently in prison, who frightened her and habitually beat her. Her mood changed frequently, showing anger and aggression, suddenly bursting into tears. She often switched between different languages: she spoke mainly in English, but she preferred her mother tongue to discuss emotionally distressing content. By the third day of admission, she seemed more alert and less fatigued. Her mannerisms had diminished. She was reading a book authored by a well-known actor and commenting on it appropriately. Although her mood was still unstable, she was more organized and started to remember and report the events leading to her admission. She reported a large consumption of different drugs (cocaine, ecstasy, and alcohol) throughout her stays on the island, and she mentioned a rave party on the day before she was hospitalized. Notably, she also reported a long-standing tendency to get into trouble or risky situations, underlining it with an ironic attitude. At the last follow-up visit, she was advised to continue her hospitalization due to her unstable condition. The toxicological report was positive for cocaine and amphetamine.

She was discharged 10 days after admission with a diagnosis of a substance-induced psychotic episode in borderline personality disorder.

### 2.2. Case 2

At around 5 a.m., a young Arab woman was brought by the police to the emergency room at the Can Misses Hospital after displaying aggressive behavior towards others, particularly tourists walking in central Eivissa. The woman was in a state of severe agitation, with poor hygiene, irregular breathing, a rapid heart rate (125 bpm), dilated pupils, and a high fever (38.5 °C). She was highly agitated and yelling incomprehensible words in both Arabic and English. She was administered intravenous diazepam, but this had no clear clinical effect. Several doses of intravenous midazolam, totaling 24 mg, were also given, but her condition did not improve: her agitation and aggressive behavior continued, and she seemed terrified, repeatedly attempting to flee. The medical team struggled to conduct an examination, as she resisted every attempt, and physical restraint was eventually necessary. However, once a female Arabic interpreter arrived, the patient calmed down. She introduced herself in fluent English and asked the men in the room to leave. She appeared hesitant to discuss the events of the previous night and was only partially aware of her surroundings: she knew she was in Europe but could not recall when she had arrived in Ibiza or who had accompanied her. During the interview, she repeatedly sat on the bed, swaying her head and shouting phrases like, “I am the daughter of God… He cast me down… I made a mistake… He no longer wants me”. She also refused to urinate, citing religious concerns about the act.

She was later transferred to the psychiatric ward, where her agitated and aggressive behavior continued, particularly towards the nursing staff. She was given an intramuscular injection of 2 mg/mL haloperidol, which helped stabilize her. A few hours later, a man claiming to be her husband arrived at the ward, requesting her discharge so they could catch a flight to Paris. Through an interpreter, the man explained that they were from Azerbaijan and had come to Ibiza for their honeymoon. They had been married two weeks earlier and were traveling through Europe, having already visited Prague and hoping to visit Paris before returning home. With the help of the interpreter, the husband agreed to postpone their flight, but he refused to provide any details about what had occurred the previous night. The patient did not meet with her husband during his initial visit.

After the haloperidol dose, the patient became more oriented in both time and space, but she remained easily distracted and continued to refuse food and drink. She was highly talkative and echolalic, with evident disturbances in her thinking process, including perseveration. Delusional thoughts with religious overtones, as well as feelings of guilt and shame, were still apparent. She could not remember her marriage and avoided any physical contact with the nursing staff and doctors. In the afternoon, approximately 12 h after her admission, her husband returned for another visit, and this time, the patient quickly threw herself into his arms. The woman, suddenly calm and quiet, was able to recall and explain who she was and why they were in Ibiza. During the rest of her hospitalization, the woman became more submissive and accepted daily talks with psychiatrists and psychologists. Despite pharmacological treatment and its progressive benefits, no recollection of the night before her admission emerged. While she continued to deny the use of psychoactive substances, urine toxicology tests showed the presence of barbiturates (methaqualone).

She was eventually discharged seven days after admission with a diagnosis of substance-induced manic episode with mixed psychotic features.

### 2.3. Case 3

A twenty-year-old boy who had been working on the island as a waiter for several years, arrived at the ER because of unusual and aggressive behaviors. He had spent the summer season at discos and parties with friends, and he had attended a rave party in Ibiza, taking several substances at the same time, during the weekend before admission. During the first interview in the ER, he appeared conscious, oriented, and cooperative, with some elements of distrust. He showed articulate speech, but the content of his thoughts was disorganized, and he seemed to lack any memory of the days before hospitalization. There was evidence of perceptual alterations, visual hallucinations, and pseudo-hallucinations, although paranoid ideation prevented a full clinical interview during the first few days. During the following days, he extensively talked about his life and family: he had adoptive parents, an adoptive sister, and three brothers. He reported previous psychological contacts during primary school because of some problems with the other children and because the teachers had complained to his parents about his restlessness and lack of attention, but his medical history appeared to be negative for previous psychiatric diagnoses. He reported daily use of cannabis and alcohol during for at least two weeks before admission, and occasional use of methamphetamine, lysergic acid diethylamide (LSD), and ketamine. He also reported recent use of an unknown liquid psychoactive substance. Talking about the latter substance, he perfectly described the hallucinatory symptoms and paranoid thoughts that occurred after taking the drug, which led to the unjustified aggressive behaviors and the subsequent hospitalization. While the visual hallucinations disappeared after a few days, auditory hallucinations, described as “a sort of music in the brain” persisted several days, as well as paranoid symptoms and aggressive behaviors The patient was discharged five days after admission, when a complete remission of symptoms was observed, with a diagnosis of a substance-induced psychotic episode. The toxicological examination was positive for cocaine and amphetamines.

### 2.4. Case 4

A 43-year-old woman from Slovakia, who had been regularly employed as a realtor in Ibiza since 2012, was accompanied to the psychiatric service of the Can Misses Hospital by her boyfriend because of externally directed aggressiveness and psychomotor excitement, which initially prevented a full clinical interview, although she did not require physical restraint. She denied any drug use or psychiatric history. During the psychological assessment conducted two days after admission, she appeared less nervous and upset with a moderate somatic anxiety, and she referred to some clinical lab results she was waiting for. She claimed to be a translator, able to speak Italian, English, and Spanish simultaneously. Her speech was pressing and unstoppable, with derailed thoughts. Disorganization, an unusual content of thoughts, and poor insight into her conditions, but no delusional symptoms or hallucinations, were observed during this first session. Over the manic excitement, she reported restlessness and insomnia, with no more than 4 h of continuous sleep per night. During the interview, she appeared hostile and suspicious, and she jumped from one topic to the other without meaningful connections between them, describing a recent discussion with her boss, the relationship with her mother, the unbearable sense of responsibility and the urge to demonstrate her moral integrity, and the ability to carry out her projects after her father’s death, but also the desire to have a child, and she reported an increased interest in sexual activities over the last number of weeks.

The day after, although more cooperative, she appeared excited, euphoric, restless, and inattentive. Her thoughts were still disorganized. She reported consumption of cocaine, amphetamines, alcohol, together with an unknown substance that someone else would have put in her drink during a rave party the day before hospitalization. Moreover, she reported increased alcohol consumption during the last 2 weeks (one bottle of wine/a day, i.e., five drinks per night per day), although she denied previous drug use. She referred to herself as a determined and headstrong woman, hiding a man inside herself, and mentioned she was working on the draft of a book about herself and the most important experiences that built her personality. She flipped through the pages while admitting that, while also promising to send me a copy of her book as soon as possible. Mannerisms and hyper-expression were clearly observed. Before the conclusion of the session, she asked to keep in touch with the interviewers. The toxicology report was positive for cocaine and amphetamine, and she was discharged one week after admission with a diagnosis of a substance-induced manic episode.

### 2.5. Case 5

A 27-year-old male was brought to the psychiatric unit by the police. A few hours earlier, the patient had reported the theft of his identity card as part of a structured and organized project made by an undefined group of persecutors. He had been working as a DJ around the island since the beginning of the summer season, with good performances and success, gaining a discrete level of popularity. His suspicions began a week before admission, during a night at one of the discos where he was used to playing, where he started to feel observed, perceiving a sense of menace and threat, which required him to interrupt his performance and return to his house. However, the same feeling reoccurred once at home, where he felt threatened and persecuted by the people sharing the apartment with him. When he woke up the next morning and he found a girl in his bed, hypothesizing that she had been placed there in order to cause him trouble with his wife, he decided to escape from his own house and hide close to a bus stop at first, and then moving to different hotels on the island, until his referral to the police. In the psychiatric unit, he was admitted in a forced regimen for the first few days. In fact, he was aggressive and extremely suspicious, presenting paranoid ideation and delusions of poisoning, and as such he refused food, medications, and any other supplies, as well as any contact with the staff. After the first two days of antipsychotic treatment with haloperidol, he gradually improved, allowing for more structured interviews and assessments. He reported a frequent use of large amounts of cocaine and other stimulants. The night of the psychotic breakdown, he remembered consuming cocaine, without recognizing its effects. He also reported to be in treatment for alcohol and cocaine use disorders, with a regular intake of topiramate and disulfiram. During the time he spent at the hospital, he showed increasing levels of insight: he started criticizing some memories, feelings, and thoughts, admitting they were difficult to believe, and probably impossible in their nature or at least in some of their elements. He also hypothesized the specific role of the substance he took the night of the psychotic onset as one of the causes of his breakdown. However, his suspiciousness continued to emerge, tapering the possibility of a clear critique of his beliefs. The toxicology report was positive for cocaine. He was eventually discharged with a diagnosis of substance-induced psychotic episode 72 h after hospitalization, when psychotic symptoms disappeared after symptomatic treatment with haloperidol and loxapine.

## 3. Discussion

### 3.1. NPS and Psychopathological Consequences

As previously reported [34,35], the participants of this case series showed significant and severe psychopathology related to psychoactive substances, as measured by the psychometric scales administered during the hospital stay.

As expected, positive symptoms as assessed by the Positive and Negative Syndrome Scale (PANSS) were more severe, showing higher scores than negative symptoms, which is consistent with previous reports [36,37]. Hallucinations, especially visual hallucinations, and other perceptual disturbances were frequently reported upon admission, confirming the hypothesis that substance-induced psychotic disorders are often associated with visual symptoms [38]. This could be explained by the powerful interaction exerted on 5HT receptors by modern hallucinogens and other substances, such as the 3,4-methylenedioxymethamphetamine (MDMA) and other similar compounds. These data are consistent with other studies on NPSs and hallucinogens, which significantly impair human perception and change the perception/movement cycle [39], affecting both critical thinking and judgement. Chemical delusions may be consequences of the intense changes drug users experience in the relationship with reality based on perceptual distortions. Indeed, although delusions are generally fixed beliefs that cannot be modified, chemical delirium is often characterized by interpretations of reality, not by revelations and/or fantastic contents [40]. Also, the delusions experienced by patients using NPSs are similar to paraphrenic delusions and related to feelings of unreality, but the ability to understand and analyze feelings and perceptions is preserved. In this regard, the lysergic psychoma model could be an interesting hypothesis [41]. Among the psychiatric manifestations observed in our sample, psychotic and mood symptoms prevailed [42]. It is important to highlight that some patients experienced disorientation in both space and time. These temporary cognitive disturbances are uncommon in psychiatric conditions, except for dissociative disorders, which are often linked to substance use [43]. Both quantitative and qualitative disturbances of consciousness should be carefully evaluated in patients suspected of alcohol or substance use. These alterations can aid in differentiating between a substance-induced psychotic state and a typical psychotic onset unrelated to substance use. Among the qualitative disturbances, the twilight state may represent a transient phase between a substance-induced psychotic episode and fully developed psychosis [40]. In this state, vigilance does not diminish, and the patient can still perform goal-directed and oriented movements. Additionally, the field of consciousness can expand and shift rapidly. At the opposite end, states of consciousness referred to as crepuscular or auroral states are classical conditions where the field of consciousness narrows, focusing on very limited content [40]. These states tend to foster illusions and hallucinations of a visionary nature. As they fade, objects lose focus and retreat into the background, and they are often replaced by real hallucinations. Depersonalization, whether experienced externally or internally, and derealization are common and reversible phenomena in these states [38,40]. Psychosis arising from the twilight condition of addictive experiences does not follow the patterns of schizophrenia, melancholia, or mania: it manifests purely as psychosis, with alterations in both cognitive and ideational realms, affecting mood and perception, though remaining in an undifferentiated state without solidifying into a specific form. In this state, the emotional spectrum intersects with schizophrenic features [40]. After this phase, it is possible for the individual’s consciousness to return to the pre-substance state. In some cases, however, this expansion of consciousness leads to a new interpretation of the experience, creating a delusional reality disconnected from previous experiences.

Our studies are in line with other research that has analyzed the symptomatology induced by NPSs [14,36,37,38,42], and more specifically, by club drugs [31].

### 3.2. NPS Users and Psychiatric Comorbidities

The majority of the individuals involved in this case series had previously been hospitalized or had undergone outpatient treatment in a psychiatric environment. This suggests that the likelihood of developing psychiatric symptoms following recent substance use is higher among those with a prior history of psychiatric and Substance Use Disorders. However, a significant portion of the sample had no reported psychiatric history. These findings indicate that the psychopathological effects of illicit substances are substantial, even in individuals without any prior psychiatric symptoms.

Also, the majority of the included subjects reported previous drug use, and only one case was confirmed as a first-time use. According to Bellis et al. [32], 7.2 percent of British tourists and 8.6 percent of Spaniards in Ibiza had tried MDMA for the first time while visiting the island, but only a much smaller percentage of Germans (1.8 percent) and people visiting Majorca (0.8, 1.5, and 1.2 percent, respectively) reported their first use of drugs while on vacation [32]. However, in our sample, the ‘holiday’ pattern of drug use on the island appeared to be different from the pattern of use reported ‘at home’. Specifically, many individuals reported drug consumption on five or more occasions per week while on vacation, while they reported limiting drug access to fewer occasions while at home [33].

Given these clinical observations, we hypothesized certain factors, such as (i) high potency substances, (ii) the quantity of consumption, and (iii) the frequency of use, may play a role triggering psychiatric symptoms in drug users. Also, we hypothesized two possible pathways through which substances can induce clinically relevant psychiatric symptoms:

1. Previous psychiatric history, personality characteristics, and traumatic load may represent a point of vulnerability that, when interacting with substances, may lead to a major problem, both in the area of mood and thinking;
2. The short-term consumption of large quantities or potent substances may contribute to the onset of psychotic symptoms or mood-independent effects.

The potential for developing a serious, long-term psychiatric disorder—such as schizophrenia, mood disorders, or psychotic disorders—following substance use remains a topic of discussion. A recent large-scale prospective study demonstrated that nearly one-third of individuals who experienced an acute psychotic reaction to substance use went on to develop schizophrenia or bipolar disorder within five years [44]. The ability of these substances to induce de novo psychosis in vulnerable individuals [14,45], which may then evolve into full-blown schizophrenia or other psychotic spectrum disorders [46], or long-lasting substance-induced psychoses, known as SREP (Substance-Related Exogenous Psychosis) [47], or lead to a full recovery, remains particularly interesting and worthy of further investigation.

Another significant aspect noted in this case series was the high level of aggression observed upon hospital admission. Whether or not patients presented with mood or purely psychotic symptoms, aggression seemed to transcend specific diagnoses, likely reflecting an inherent feature related to substance use [47]. This raises concerns about the heightened risk of workplace violence faced by psychiatric staff, especially nurses and nursing assistants, as shown in a recent study from Italy [48]. The increasing presence of NPSs (new psychoactive substances) in custodial environments is also an issue [49].

Binge drinking and sleep deprivation emerged as common factors in nearly all individuals assessed. The data on binge drinking align with existing research, which identifies binge drinking as a widespread pattern of alcohol consumption, particularly among adolescents and young adults [50]. Despite the rising use of NPSs across Europe [9,51], with associated reports of severe psychopathological outcomes and fatalities [31,34,50], alcohol, cocaine, and first-generation psychedelics still constitute the primary substances involved in both direct and indirect deaths. However, more advanced methods for analyzing these substances in post-mortem cases need further investigation [31,52]. If the increasing number of deaths is not solely attributed to the direct use of these highly toxic substances, alternative hypotheses need to be considered:

1. Could there be a broader base of substance users, increasing the number of people at risk due to predisposing factors? [2]
2. Is there a potential involvement of lifestyle and behavioral factors, such as patterns of poly-substance use, binge drinking, intoxication, and strenuous physical activities at rave parties? [4,37]
3. Is it possible that the pursuit of boundaries explored by “psychonauts” online could extend into real-life trends?

### 3.3. Study Limitations

This article is primarily based on case reports, with a small study sample and selection biases, making it difficult to generalize the findings. Furthermore, inconsistencies in the purity and composition of street drugs marketed as NPSs complicated the identification of the specific substances and dosages involved, further hindering the generalization of the results. Given that these are case reports with predominantly psychopathological findings, a statistical analysis could not be performed.

## 4. Methods

All participants were admitted to the Psychiatry Department of the Can Misses Hospital during the summer nightclub seasons between May 2015 and October 2016. This sample was drawn from a larger cohort of 223 individuals transferred to the hospital emergency room from discotheques and other nightlife venues across the island, presenting with signs of intoxication or psychiatric symptoms of interest. Each patient underwent an evaluation according to the DSM-5 diagnostic criteria. Inclusion criteria required subjects to be between the ages of 18 and 75 and to be experiencing intoxication related to psychoactive substances. Patients were excluded if they had conditions such as delirium tremens, epilepsy, hepatic encephalopathy, dementia, or other neurological disorders, along with severe cardiac failure, diabetes mellitus, significant liver impairment, renal failure, or neoplastic diseases, which were ruled out through clinical assessment.

Structured interviews were conducted after the intoxication symptoms had subsided during hospitalization, collecting demographic information (age, gender, family status, and nationality) and socioeconomic details (living situation, employment status, and education level). The interview explored both recent and past medical and psychiatric histories, as well as patterns of alcohol and substance use, including habits related to tobacco, caffeine, cannabis, cocaine, and heroin, with a special focus on new psychoactive substances (NPSs). Specific inquiries were made about the recent and lifetime use of synthetic cannabinoids, synthetic cathinones (such as mephedrone, methylone, methylenedioxypyrovalerone (MDPV), and alpha-pyrrolidinopentiophenone (a-PVP)), amphetamines and methamphetamines, plant-based substances (like ayahuasca, kratom, and Salvia divinorum), GHB and GBL (gamma-butyrolactone), dissociatives (e.g., ketamine and methoxetamine), and psychedelics (such as LSD and psilocybin-containing mushrooms). Misuse of prescription medications, including benzodiazepines, methylphenidate, and opioid analgesics, was also examined.

The data collection adhered to principles of anonymity and confidentiality. All participants were fully briefed on the study design, and written informed consent was obtained from each subject in accordance with the Declaration of Helsinki. Comprehensive data for the broader study group can be found in the referenced sources [31,34,35].

Ethical approval was granted by the University of Hertfordshire Health and Human Sciences ECDA, under protocol no. aPHAEC1042(03); by the CEI Illes Balears, protocol no. IB 2561/15PI (“Evaluation and Assessment of Substance and Alcohol-Induced Symptoms”, Dr. Cristina Merino del Villar, Area de Salud Mental de Eivissa y Formentera); and by the University “G. d’Annunzio” of Chieti-Pescara, protocol no. 7/09 04-2015.

## 5. Conclusions

This series of cases highlighted the significant usage of psychoactive substances in specific recreational contexts, often involving traditional drugs, NPSs, which resulted in severe and sometimes long-lasting psychological effects. Symptoms included mania, heightened positivity, and altered states of consciousness. Aggressive behavior was notably increased among individuals who primarily used cannabinoids. The management of Novel Psychoactive Substances (NPSs) requires a multi-faceted strategy. Legislation must include flexible drug scheduling laws to rapidly adapt to emerging substances, while enhancing real-time monitoring and international collaboration to combat global NPS distribution [53]. Public education campaigns are essential for raising awareness, and healthcare providers require specialized training for NPS-related incidents. Online platforms must be monitored in partnership with tech companies to regulate NPS promotion [54].

The European Union and the United States have implemented risk-based approaches, such as temporary bans and substance categorization, to improve detection and control. Global efforts led by the UNODC aim to standardize regulations and foster cooperation. Despite these actions, continuous improvements in legislation, education, and international collaboration are essential to address the evolving threats of NPSs and protect public health [54,55].

The findings of this study underscore the need for in-depth research and close surveillance of substance misuse to develop targeted prevention measures. Enhanced monitoring and rigorous assessment are crucial to understanding the full impact and optimizing treatment protocols for those affected by these substances.

## Figures and Tables

**Table 1:** Summary table of case series.

Cases	Gender; Age	Substance	Medical/Psychiatric Comorbidity	Symptoms	Treatment	Discharge Diagnosis
Case 1	Female, 27 yrs	Simultaneously poly-abuse (cocaine, ecstasy, and alcohol)	None	Psychotic episode with delusional ideations, conceptual disorganization; paranoia; psychomotor agitation, verbal and outwardly directed aggression, and emotional lability; spatio-temporal disorientation; insomnia, lack of energy; confusion; mannerisms; and switching between languages	Olanzapine, haloperidol, and lorazepam	Substance-induced psychotic episode in borderline personality disorder
Case 2	Female, NA yrs	Methaqualone	None	Neglected in clothing and personal hygiene; agitation and aggressive behavior; genealogical/grandeur delusion with perseveration and mystic delirium; verbose and echolalic; and excessive thoughts of guilt and shame	Intravenous dose of diazepam; intravenous administrations of midazolam; and haloperidol	Substance-induced manic episode with mixed psychotic features
Case 3	Male, 20 yrs	None, only daily use of cannabis with and without alcohol in the last two weeks. Occasional use of methamphetamine, lysergic acid diethylamide (LSD), and ketamine	Alcohol, cannabis, and methamphetamine use in addition to another unknown liquid psychoactive substance	Disorganization; amnesia; perceptual alterations, visual hallucinations, and pseudo-hallucinations; and paranoia	NA	Substance-induced psychotic episode
Case 4	Female, 43 yrs	Cocaine, amphetamines, alcohol, and a probably unknown substance that someone else would have put in her drink	None	Manic episode with aggressiveness and psychomotor excitement; pressing speech with the tendency to derail thinking and speaking in three languages. Euphoria; mannerisms disorientation. Amnesia and difficulties in keeping attention	Haloperidol and loxapine	Substance-induced manic episode
Case 5	Male, 27 yrs		Alcohol and cocaine use disorders, with a regular intake of topiramate and disulfiram	Paranoid ideation and delusional about poisoning; aggressiveness and suspiciousness, manic symptoms	Haloperidol and loxapine	Substance-induced psychotic episode

NA: Not available.

## Data Availability

The data presented in this study are available on request from the corresponding author due to privacy concerns.
